# O Nível de Endocan Sérico pode ser Usado como Biomarcador para Prever Aterosclerose Subclínica em Pacientes Pré-Diabéticos?

**DOI:** 10.36660/abc.20210797

**Published:** 2022-07-28

**Authors:** Yucel Arman, Adem Atici, Ozgur Altun, Remzi Sarikaya, Sengül Aydin Yoldemir, Murat Akarsu, Orkide Kutlu, Guzin Zeren Ozturk, Pinar Demir, Mustafa Ozcan, Recep Yilmaz Bayraktarli, Tufan Tukek

**Affiliations:** 1 University of Health Sciences Prof Dr Cemil Tascioglu City Hospital Department of Internal Medicine Istanbul Turquia University of Health Sciences, Prof Dr Cemil Tascioglu City Hospital, Department of Internal Medicine, Istanbul – Turquia; 2 Istanbul Medeniyet University Goztepe Prof. Dr. Suleyman Yalcin City Hospital Department of Cardiology Istanbul Turquia Istanbul Medeniyet University, Goztepe Prof. Dr. Suleyman Yalcin City Hospital, Department of Cardiology, Istanbul – Turquia; 3 University of Health Sciences Van Education and Research Hospital Department of Cardiology Van Turquia University of Health Sciences, Van Education and Research Hospital, Department of Cardiology, Van – Turquia; 4 University of Health Sciences Istanbul Bakirkoy Dr Sadi Konuk Training and Research Hospital Department of İnternal Medicine Istanbul Turquia University of Health Sciences, Istanbul Bakirkoy Dr Sadi Konuk Training and Research Hospital, Department of İnternal Medicine, Istanbul – Turquia; 5 University of Health Sciences Kanunİ Sultan Suleiman Traİnİng and Research Hospİtal Department of İnternal Medicine Istanbul Turquia University of Health Sciences, Kanunİ Sultan Suleiman Traİnİng and Research Hospİtal, Department of İnternal Medicine, Istanbul – Turquia; 6 University of Health Sciences Sisli Hamidiye Etfal Training and Research Hospital Department of Family Medicine Istanbul Turquia University of Health Sciences, Sisli Hamidiye Etfal Training and Research Hospital, Department of Family Medicine, Istanbul – Turquia; 7 University of Health Sciences Prof Dr Cemil Tascioglu City Hospital Department of Radiology Istanbul Turquia University of Health Sciences, Prof Dr Cemil Tascioglu City Hospital, Department of Radiology, Istanbul – Turquia; 8 Istanbul University Istanbul Faculty of Medicine Department of İnternal Medicine Istanbul Turquia Istanbul University, Istanbul Faculty of Medicine, Department of İnternal Medicine, Istanbul – Turquia

**Keywords:** Aterosclerose, Espessura Íntima-Media Carotídea, Estado Pré-Diabético

## Abstract

**Fundamento:**

Pacientes pré-diabéticos têm um risco aumentado de doença cardiovascular aterosclerótica, e, portanto, a detecção precoce é importante.

**Objetivo:**

Nosso estudo teve o objetivo de revelar a usabilidade dos níveis de endocan sérico como biomarcador no diagnóstico de aterosclerose subclínica em pacientes pré-diabéticos, com base em medições de EIMC.

**Métodos:**

Os participantes foram classificados de acordo com a presença (n=42) ou ausência (n=42) de pré-diabetes. Os valores de endocan sérico, glicemia em jejum, insulina em jejum e hemoglobina glicada (HbA1c) dos pacientes foram examinados e a EIMC foi medida. O nível de significância para a análise estatística foi 0,05.

**Resultados:**

Apesar de se ter determinado que os níveis de endocan sérico são mais baixos em pacientes pré-diabéticos em comparação com o grupo de controle (p=0,042), determinou-se que os valores de EIMC são mais altos (p=0,046). A avaliação do endocan sérico por análise regressiva multivariada detectou que seu nível estava associado à EIMC, independentemente de outros parâmetros (p=0,007). Encontramos uma correlação negativa entre insulina plasmática em jejum e níveis de endocan (r=-0,320, p=0,001).

**Conclusões:**

Este estudo demonstrou que a espessura íntima-média de carótida é mais alta e o nível de endocan sérico é mais baixo em pacientes pré-diabéticos. Os níveis de endocan sérico diminuídos em pacientes pré-diabéticos podem ser um fator que contribui para os mecanismos de formação de aterosclerose.

## Introdução

O pré-diabetes, definido como os níveis entre índices glicêmicos normais e diabéticos, está aumentando rapidamente em todo o mundo. Aproximadamente 38% da população adulta no Estados Unidos da América^1^ e cerca de 50% da população chinesa têm pré-diabetes.^2^ O pré-diabetes é importante devido ao aumento do risco de complicações microvasculares e macrovasculares e o avanço para o diabetes tipo 2 em um curto período. É sabido que os altos níveis de glicemia plasmática são um importante fator de risco de doença cardiovascular aterosclerótica.^3^ Além disso, a resistência à insulina pode estar associada à aterosclerose devido a piores perfis lipídicos,^4^ estado pró-inflamatório^5^ e disfunção endotelial.^6^

A detecção de doença cardiovascular aterosclerótica nesse período inicial é importante para o acompanhamento e o tratamento. A espessura íntima-média da carótida (EIMC) é usada para detectar aterosclerose subclínica nos estágios iniciais e demonstrou prever eventos cardiovasculares.^7 - 10^ Cada 0,1 mm de aumento na EIMC aumenta o risco de infarto do miocárdio em 10-15% e de acidente vascular cerebral em 13-18%.^11^ Ela é muito apropriada para uso em estudos populacionais de grande escala, pois é não invasiva e pode ser obtida com uma medição simples.

Além de métodos não invasivos para determinar o desenvolvimento da aterosclerose, sabe-se que vários biomarcadores também são incluídos nas previsões. A molécula-1 específica da célula endotelial (ESM-1), chamada de endocan, é um proteoglicano liberado pelas células endoteliais sob o controle de citocinas inflamatórias. O endocan ativa compostos garantindo o substrato necessário para coleta, adesão e transmigração de leucócitos no endotélio ativado.^12^ Estudos anteriores determinaram que os níveis de endocan sérico eram maiores em pacientes com diabetes tipo 2 e síndrome coronária aguda em comparação com os grupos de controle.^13 , 14^ Estudos demonstraram que os níveis de endocan sérico estavam associados à gravidade da doença.^10 - 12^

Existem estudos que avaliam os níveis de endocan sérico em pacientes pré-diabéticos e com resistência à insulina. Entretanto, não é claro se as alterações nos níveis de endocan sérico são uma causa ou uma consequência, especialmente no caso de eventos ateroscleróticos. Quando os níveis de endocan sérico foram comparados entre grupos de pacientes e grupos de controle, diferentemente dos valores altos do diabetes tipo 2, determinou-se que eles eram baixos ou inalterados no grupo de pré-diabéticos.^15 , 16^ Embora a tendência a aterosclerose aumente nos pacientes pré-diabéticos e diabéticos, as diferenças nos níveis de endocan sérico são notáveis. Existem estudos que avaliam os níveis de endocan nos eventos ateroscleróticos e vasculares em pacientes com diabetes tipo 2. Entretanto, não foi possível encontrar nenhum estudo na literatura que avaliasse os níveis de endocan em pacientes pré-diabéticos com aterosclerose.

Nosso estudo teve o objetivo de revelar o papel dos níveis de endocan sérico na previsão de aterosclerose subclínica baseado em pacientes pré-diabéticos em EIMC.

## Métodos

Nosso estudo está em conformidade com a Declaração de Helsinki e foi aprovado pelo comitê de ética em pesquisa do hospital Prof Dr Cemil Tascioglu City (aprovação número 525). Os participantes assinaram um termo de consentimento informado. Este estudo transversal foi realizado no ambulatório de medicina interna de nosso hospital de cuidado terciário entre junho e agosto de 2021. Foram incluídos no estudo 84 participantes, com mais de 18 anos de idade, dos quais 42 eram pacientes pré-diabéticos e 42 eram normoglicêmicos (o IMC, a idade e o sexo eram semelhantes).

De acordo com os critérios da *American Diabetes Association* (ADA), pessoas com índices glicêmicos em jejum entre 100-125 mg/dL (tolerância à glicose prejudicada (IGT)) ou HbA1c 5,7-6,4%, ou níveis de glicemia plasmática de 2 horas durante o teste oral de tolerância à glicose (TOTG) a 75 g entre 140 e 199 mg/dL (tolerância à glicose prejudicada (IGT)), foram incluídas no grupo dos pré-diabéticos.^17^ Participantes normoglicêmicos com valores mais baixos foram incluídos no grupo de controle. Os indivíduos nos grupos normoglicêmico e pré-diabético não estavam fazendo uso de medicamentos antidiabéticos.

Indivíduos com histórico de infarto do miocárdio ou revascularização coronária, eventos cerebrovasculares, diagnóstico prévio de doença cardiovascular ou insuficiência cardíaca sistólica, doença valvar grave, cardiomiopatia hipertrófica, angina pectoris, variações da onda ST-T no eletrocardiograma, ondas Q, bloqueio do ramo esquerdo, doença hepática ou renal crônica, malignidade ativa, hipertensão, doenças inflamatórias, doença do sistema respiratório, doença arterial periférica, tabagismo, ou que se recusaram a participar, foram excluídos do estudo.

A pressão arterial dos participantes foi medida, e o IMC foi calculado medindo sua altura e peso (peso/altura ao quadrado, kg/m^2^). Depois de jejum noturno, foram analisados glicemia, insulina HbA1c, níveis lipídicos (colesterol de lipoproteína de alta densidade (HDL-C), colesterol de lipoproteína de baixa densidade (LDL-C) e triglicérides), proteína C-reativa (PCR), creatinina, e endocan sérico. Os valores do modelo de avaliação da homeostase da resistência à insulina (HOMA-IR) foram calculados com a fórmula, (glicemia em jejum x níveis de insulina em jejum)/405.

### Medições de endocan sérico

Após o jejum noturno, foram coletados 10 ml de sangue venoso dos participantes. As amostras foram centrifugadas por 10 minutos a 1700 rpm. O soro foi armazenado a -80 ℃ até a análise. Os níveis de endocan sérico foram medidos com um kit de ensaio de imunoabsorção enzimática (ELISA) de acordo com o protocolo do fabricante (Human Endocan Elisa Kit; lote nº: 201506, Nº cat.: E3160Hu, Sunred Biological Technology, Xangai, China). Os resultados são apresentados em ng/L. O intervalo de medição do kit é 31-2000 ng/L.

### Avaliação da espessura da íntima-média da artéria carótida

A EIMC foi medida usando-se um transponder de gama linear multifrequência (12 MHz) (Samsung HS50 GE Ultrasound). Todas as medições foram feitas em imagens de modo B de alta resolução. Para as medições de EIMC, os pacientes foram colocados em posição supina com a cabeça virada a 45º na direção oposta ao lado da medição. Imagens de modo B da extensão do segmento distal da artéria carótida principal direita foram obtidas para três seções em sequência da parede mais distante da artéria carótida principal. Em seguida, a distância entre as interfaces entre sangue-íntima e média-adventícia foi medida para cada seção. A EIMC foi calculada pela média dos valores das medições.

### Análise estatística

As análises estatísticas foram realizadas utilizando-se o software SPSS, versão 26.0 (SPSS Inc., Chicago, Illinois, EUA). Média e desvio padrão foram usados para variáveis contínuas com distribuição normal, e mediana e faixa interquartil foram usadas para as sem distribuição normal. Variáveis categóricas são expressas como números absolutos e porcentagens. A distribuição das variáveis foi avaliada com o teste Kolmogorov–Smirnov. As variáveis contínuas foram comparadas usando-se o teste T (não pareado) de duas amostras independentes ou o teste U de Mann-Whitney, de acordo com sua distribuição. O teste qui-quadrado foi usado para variáveis categóricas. Os testes de Pearson ou Spearman foram usados para análise de correlação dependendo se as variáveis eram paramétricas ou não paramétricas. A análise regressiva linear multivariada foi usada para avaliar as determinantes da EIMC. A distribuição normal de todos os parâmetros é necessária para a análise regressiva linear multivariada. Obtivemos a distribuição normal a partir dos logaritmos do endocan sérico e dos níveis de triglicérides. O nível de significância estatística foi definido em p <0,05.

### Reprodutibilidade

Considerando que a concordância entre intraobservador e interobservador é 0,75, o tamanho mínimo da amostra (considerando Erro de tipo de 0,05, Erro de tipo III de 0,20 e Poder de 0,80) é n=13. Considerando as possibilidades de perdas por qualquer motivo, 15 pessoas foram incluídas no estudo.

### Análise de poder

A análise de poder foi realizada com o programa G-power. Com base em dados anteriores na literatura, para tamanho de efeito 0,57, a parcela de 5% do erro alfa e o poder de 80% de representar a população, o menor tamanho para cada grupo amostral foi calculado como 39.

## Resultados

Idade, sexo e valores de IMC dos grupos pré-diabético e normoglicêmico foram semelhantes (p>0,05).

Os níveis de endocan sérico foram significativamente mais baixos no grupo pré-diabético do que no grupo de controle (p=0,042), e os valores de EIMC foram mais altos (p=0,046) ( Tabela 1 ).

**Tabela 1:** Características demográficas e achados laboratoriais em pacientes pré-diabéticos e de controle

	Grupo de controle n=42	Grupo de pacientes pré-diabéticos n=42	p
Idade (anos)	47,8±9,7	49,9±8,5	0,112
Sexo (F/M)	28/14	30/12	0,814
IMC (kg/m^2^)	33,8±4,1	32,2±8,8	0,066
Endocan (ng/L) *	138 (84-300)	120 (65-185)	0,042
FPI (µU/ml)	11,2±5,3	20,1±8,8	<0,001
FPG (mg/dL)	87±5,3	103±9,7	<0,001
2-h GP durante 75-g TOTG (mg/dL)	101±19	141±34	<0,001
HOMA-IR	2,4±1,1	5,2±2,3	<0,001
HbA1c (%)	5,5 ±0,3	5,9±0,5	0,039
PCR (mg/dL)	4,9± 2,6	5,1±2,9	0,245
Colesterol total (mg/dL)	188±32	206±33	0,020
Colesterol LDL (mg/dL)	110±31	120±26	0,107
Colesterol HDL (mg/dL)	53±11	49±13	0,103
TG (mg/dL)*	108 (79-133)	152 (95-257)	0,002
EIMC (mm)	0,67±0,16	0,74±0,17	0,046

*IMC: índice de massa corporal; FPI: insulina plasmática em jejum; FPG: glicemia plasmática em jejum; GP: glicemia plasmática; TOTG: teste oral de tolerância à glicose; HOMA-IR: modelo de avaliação da homeostase da resistência à insulina; HbA1c: hemoglobina glicada; PCR: proteína C reativa; LDL: lipoproteína de baixa densidade; HDL: lipoproteína de alta densidade; TG: triglicérides; EIMC: espessura íntima-média da carótida.*

**Figura 1:**
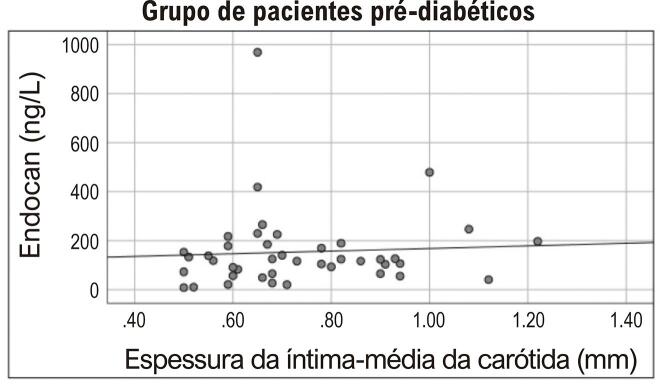
Correlação entre níveis de endocan plasmático e valores de EIMC no grupo de pacientes pré-diabéticos. (r=0,104, p=0,514)

**Figura 2:**
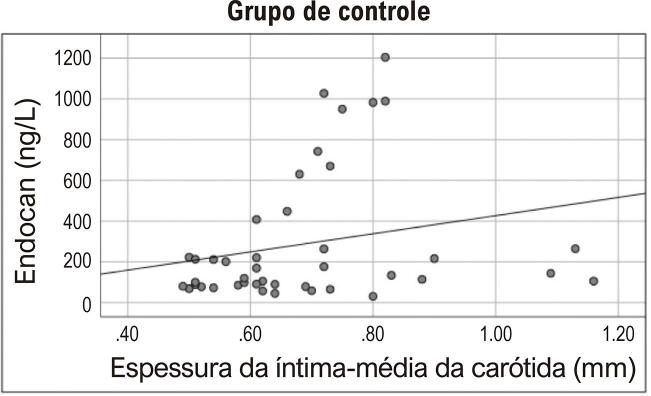
Correlação entre níveis de endocan plasmático e valores de EIMC no grupo de controle (r=0,340, p=0,028)

Houve uma correlação significativa entre o valor de EIMC e idade e níveis de triglicérides de todos os participantes ( Tabela 2 ). A análise regressiva linear multivariada de idade, endocan, HbA1c, FPI, FPG, e valores de triglicérides foi realizada com EIMC. O logaritmo dos valores de endocan sérico e triglicérides foram obtidos para garantir a distribuição normal. Detectou-se que o nível de endocan sérico estava associado à EIMC, independentemente de outros parâmetros (p=0,007) ( Tabela 3 ). Apesar de não haver correlação entre os níveis de endocan sérico e as medições de EIMC no grupo pré-diabético (r=0,104 p=0,514) ( Figura 1 ), foi encontrada uma correlação positiva no grupo sem pré-diabetes (r=0,340, p=0,028) ( Figura 2 ).

**Tabela 2:** Correlações entre EIMC e outros parâmetros

	Todos os participantes (n=84)		Grupo de controle (n=42)		Grupo de pacientes pré-diabéticos (n=42)	
	
	r	p	r	p	r	p
Endocan (ng/L) *	0,206	0,060	0,340	0,028	0,104	0,514
Idade (anos)	0,363	0,001	0,490	0,001	0,215	0,172
IMC (kg/m^2^)	-0,015	0,895	-0,009	0,956	0,034	0,833
FPI (µU/ml)	0,180	0,104	0,360	0,021	0,020	0,900
FPG (mg/dL)	0,195	0,075	0,212	0,178	0,119	0,454
2-h GP (TOTG)	0,166	0,131	0,080	0,485	0,164	0,300
HOMA-IR	0,180	0,102	0,379	0,013	0,004	0,982
HbA1c (%)	0,242	0,080	0,349	0,143	0,199	0,260
PCR (mg/dL)	0,077	0,520	0,063	0,694	0,065	0,730
C Total (mg/dL)	-0,015	0,895	-0,076	0,632	-0,015	0,927
LDL-C (mg/dL)	-0,031	0,781	-0,093	0,557	-0,192	0,223
HDL-C (mg/dL)	-0,111	0,313	0,032	0,839	0,227	0,149
TG (mg/dL)*	0,257	0,018	0,306	0,030	0,342	0,027

IMC: índice de massa corporal; FPI: insulina plasmática em jejum; FPG: glicemia plasmática em jejum; GP: glicemia plasmática; TOTG: teste oral de tolerância à glicose; HOMA-IR: modelo de avaliação da homeostase da resistência à insulina; HbA1c: hemoglobina glicada; PCR: proteína C reativa; C Total: colesterol total; LDL-C: colesterol de lipoproteína de baixa densidade; HDL-C, colesterol de lipoproteína de alta densidade; TG: triglicérides; EIMC: espessura íntima-média da carótida. *Teste de correlação de Spearman, outros: Teste de correlação de Pearson.

**Tabela 3:** Análise regressiva linear multivariada mostrando os preditores de EIMC

	Beta	IC 95%		p

		Inferior	Superior	
Idade	0,525	0,004	0,016	0,002
FPI	0,324	-0,001	0,016	0,068
Log TG	-0,142	-0,381	0,154	0,396
Log (Endocan)	0,435	0,056	0,336	0,007
HbA1c	0,181	-0,053	0,219	0,222
PCR	0,024	-0,019	0,022	0,862

*FPI: insulina plasmática em jejum; Log: logaritmo; TG: triglicérides; HbA1c: hemoglobina glicada; PCR: proteína C reativa.*

**Figura 3:**
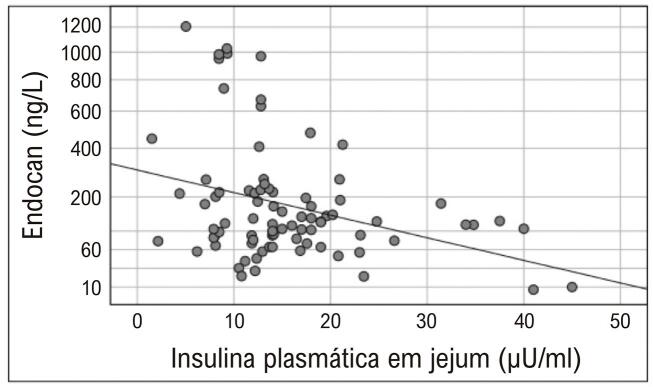
Correlação entre níveis de endocan sérico e insulina plasmática em jejum (r=-0,320, p=0,001)

As correlações entre os parâmetros da Tabela 1 e os níveis de endocan sérico foram examinadas. Desses parâmetros, apenas a insulina em jejum foi correlacionada aos níveis de endocan. Essa correlação foi negativa (r=-0,320, p=0,001) ( Figura 3 ).

### Reprodutibilidade

Um total de 15 pacientes foram selecionados aleatoriamente para análise de variabilidade inter- e intraobservador. A compatibilidade entre os valores de EIMC intra- e interobservador foi calculada. Os coeficientes de correlação intraclasse para variabilidade intraobservador e interobservador foram, respectivamente: 0,93 (IC 95%, 0,87–0,97) e 0,90 (IC 95%, 0,85–0,95) para EIMC.

## Discussão

Nosso estudo teve o objetivo de explicar o papel dos níveis de endocan na previsão da aterosclerose subclínica em pacientes pré-diabéticos com base em medições de EIMC. Os níveis de endocan plasmático foram mais baixos no grupo de pacientes pré-diabéticos do que no grupo de controle. Em contraste, os valores de EIMC foram mais altos em pacientes pré-diabéticos. Em nosso estudo, não houve correlação entre valores de EIMC e níveis de endocan sérico. Quando os grupos foram avaliados separadamente, a correlação entre medições de EIMC e níveis de endocan foi detectada no grupo normoglicêmico, mas não no grupo pré-diabético. Entretanto, dependendo dos resultados da análise regressiva, os níveis de endocan sérico explicaram significativamente o valor de EIMC.

Muitos estudos mostram que o pré-diabetes pode causar doenças cardiovasculares.^3 - 6^ Além disso, a carga de aterosclerose coronária em pacientes pré-diabéticos é mais alta do que em pessoas normais. Especificamente, a carga de aterosclerose precede os sintomas de diabetes tipo 2. Em nosso estudo, os valores de EIMC foram altos em pacientes pré-diabéticos, o que é compatível com estudos usando a EIMC como marcador de aterosclerose subclínica.^18 , 19^

Pacientes pré-diabéticos têm hiperinsulinemia devido à resistência à insulina, e os resultados de nosso estudo são compatíveis com isso. Uma correlação negativa foi confirmada entre insulina plasmática em jejum e níveis de endocan. Pode-se dizer que os níveis de endocan sérico são baixos em pacientes pré-diabéticos devido o estado hiperinsulinêmico.

A relação entre hiperinsulinemia e aterosclerose foi demonstrada por estudos anteriores. A resistência à insulina despertou grande interesse nas comunidades médica e científica devido a sua associação a doenças cardiovasculares. Entretanto, o mecanismo molecular que liga a resistência à insulina ao desenvolvimento e/ou avanço da aterosclerose continua a ser um enigma. Alguns mecanismos se destacam em relação a essa situação. A sinalização de insulina desempenha um papel crítico na ativação da sintase de óxido nítrico, que regula a produção de óxido nítrico.^20 , 21^ O óxido nítrico é um vasodilatador e um agente antiaterogênico potente.^20^ A deficiência de óxido nítrico ativa várias vias envolvidas na aterogênese.^22 , 23^ Portanto, um defeito na sinalização de insulina além de prejudicar a utilização da glicose também causa hipertensão e aterosclerose acelerada. É difícil distinguir o efeito da resistência à insulina da hiperinsulinemia compensatória que sempre a acompanha. Já se sugeriu que, se o efeito prejudicial da resistência à insulina é resultado da diminuição da ação da insulina, a hiperinsulinemia compensatória pode ser apenas um observador inocente. Inversamente, se certos aspectos da ação da insulina não são afetados pela diminuição da potência da insulina, a presença de hiperinsulinemia compensatória pode ter seu efeito próprio. Consequentemente, a hiperinsulinemia compensatória pode estimular ou até superestimular certos aspectos da ação da insulina em várias células e tecidos. Portanto, o ponto crítico no entendimento do papel da resistência à insulina é determinar se a ação reduzida da insulina (efeito da resistência à insulina) coexiste com a ação normal ou até aumentada da insulina (efeito da hiperinsulinemia) dentro do mesmo tecido e da mesma célula. Essa tarefa é possibilitada pela revelação da cadeia de sinalização intracelular da insulina. A hiperinsulinemia é um fator de crescimento potente,^24 - 28^ cujos efeitos de promoção de crescimento são mediados pela via da proteína quinase ativada por mitógenos (MAP).^29^ Após a interação entre o substrato 1 do receptor de insulina (IRS-1) e proteína transformadora contendo domínios com homologia a Src 2 (SH2), a quinase regulada por sinal extracelular (ERK) é ativada,^30 , 31^ transloca-se para o núcleo e catalisa a fosforilação de fatores de transcrição que promovem o crescimento celular, a proliferação celular e a diferenciação celular.^30^ Portanto, essa via tem um papel importante na aterogênese.

Além de sua função nos mecanismos ateroscleróticos, já se relatou que a insulina atenua a resposta inflamatória sistêmica induzida por endotoxina diminuindo a expressão do TNF-α e aumentando a cascata anti-inflamatória.^26 , 32^ A expressão do endocan é regulada diferencialmente por citocinas. O TNF-α e a interleucina-1 beta (IL-1β) regula para cima e o interferon-gama (IFN-γ) regula para baixo a secreção do endocan.^33^ O efeito redutor da hiperinsulinemia no TNF-α pode explicar a diminuição dos níveis de endocan sérico. Além disso, Janke et al. demonstraram que o endocan é expresso por adipócitos humanos e que a administração de insulina reduz a produção do endocan em adipócitos. Por esse motivo, já se sugeriu que a secreção de endocan por adipócitos pode afetar significativamente os níveis de endocan locais ou sistêmicos.^34^ Em nosso estudo, o efeito supressor da insulina nos adipócitos pode ser outro fator efetivo nos baixos níveis de endocan plasmático no grupo de pacientes pré-diabéticos.

Menon et al. pesquisaram o papel do endocan durante a formação de lesão aterosclerótica em ratos homozigotos para ApoE e identificaram altos índices de expressão de placas ateroscleróticas. No estudo, a expressão de endocan tinha níveis baixos no endotélio quiescente, ao mesmo tempo em que se mostrava regulada para cima no endotélio ativado.^35^ Os sujeitos em nosso grupo de estudo foram selecionados entre pessoas sem doença vascular conhecida ou qualquer outra situação que causasse inflamação. Por esse motivo, há uma probabilidade alta de que tanto os sujeitos do grupo de controle quanto os pacientes pré-diabéticos tinham endotélio quiescente. Nesse caso, pode-se dizer que, em nosso grupo de pacientes, o efeito da aterosclerose subclínica na secreção de endocan pelo endotélio pode ser limitado. Consideramos que o efeito da insulina no TNF-α e tecido adiposo é mais dominante e causa uma diminuição do nível de endocan sérico.

Já se demonstrou que os níveis de endocan plasmático aumentam dependendo da gravidade da doença em pacientes com aterosclerose, inflamação vascular e síndrome coronária aguda. Determinou-se que o aumento no nível de endocan sérico está associado com doenças cardíacas ateroscleróticas, mas um valor de corte ainda não foi determinado.^36 , 37^ Esse aumento no nível de endocan sérico é aceito como preditor de aterosclerose em muitos estudos. Já foi sugerido que o endocan seja um inibidor funcional do antígeno 1 associado à função do linfócito (LFA-1) e da interação com a molécula de adesão intercelular-1 (ICAM-1), sugerindo seu papel anti-inflamatório, pela inibição do rolamento, da adesão ou da transmigração de leucócitos.^12^ O efeito benéfico obtido *in vivo* pelo bloqueio da adesão a mAbs em camundongos e em outros modelos animais demonstra claramente que o LFA-1 e a ICAM-1 estão envolvidos em inflamação aguda,^38^ lesão por isquemia/reperfusão,^39^ rejeição de aloenxerto^40 - 42^ e imunidade antitumoral. Portanto, pode-se dizer que o endocan é secretado do endotélio em resposta a inflamação aguda e desempenha um papel regulatório com seu efeito anti-inflamatório. Em nosso estudo, demonstramos que os níveis de endocan sérico diminuíram em pacientes pré-diabéticos, provavelmente devido à hiperinsulinemia. Podemos concluir que o endocan tem um papel inibidor na interação entre LFA-1 e ICAM-1. Um aumento na atividade da ICAM-1 é esperado com a diminuição dos níveis de endocan. O aumento da atividade da ICAM-1 pode levar à inflamação vascular. A ICAM-1 é uma molécula bem conhecida que está envolvida na patogênese da placa aterosclerótica.^43 , 44^

Em estudos com grupos sem pré-diabetes ou resistência à insulina, os níveis de endocan sérico foram elevados, possivelmente em resposta à inflamação no vaso aterosclerótico. Entretanto, nosso estudo mostrou que essa resposta era insuficiente e que os níveis de endocan sérico diminuíram em pacientes com pré-diabetes e aterosclerose, especialmente devido à hiperinsulinemia. Níveis baixos de endocan sérico podem estar envolvidos nos mecanismos de formação da aterosclerose. São necessários estudos abrangentes sobre esse assunto.

### Limitações do estudo

Há algumas limitações neste estudo. A principal limitação é o número baixo de pacientes e o fato de o estudo ser realizado em um centro único. Segundo, as medições de EIMC foram usadas ao avaliar a aterosclerose subclínica. Por fim, outra limitação é que não sabemos há quanto tempo nossos pacientes eram pré-diabéticos.

## Conclusões

Nossos resultados demonstram que a hiperinsulinemia causa uma redução nos níveis de endocan. Entretanto, não há valor-limiar para prever a aterosclerose. A redução nos valores de endocan sérico medidos periodicamente no acompanhamento dos pacientes com pré-diabetes pode dar mais informações sobre o desenvolvimento da aterosclerose. É necessário realizar estudos prospectivos para este fim.
