# Supplementary material for: O Nível de Endocan Sérico pode ser Usado como Biomarcador para Prever Aterosclerose Subclínica em Pacientes Pré-Diabéticos?
Source: Arq Bras Cardiol. 2022 Jul 28;119(4):544–50. [Article in Portuguese] doi: 10.36660/abc.20210797 (PMC9563878; doi:10.36660/abc.20210797)
Supplement: Translation

# Translation: Can the Serum Endocan Level Be Used as a Biomarker to Predict Subclinical Atherosclerosis in Patients with Prediabetes?

**Authors:** Yucel Arman, Adem Atici, Ozgur Altun, Remzi Sarikaya, Sengül Aydin Yoldemir, Murat Akarsu, Orkide Kutlu, Guzin Zeren Ozturk, Pinar Demir, Mustafa Ozcan, Recep Yilmaz Bayraktarli, Tufan Tukek

**Affiliations:** 1 Turquia University of Health Sciences, Prof Dr Cemil Tascioglu City Hospital, Department of Internal Medicine, Istanbul – Turquia; 2 Turquia Istanbul Medeniyet University, Goztepe Prof. Dr. Suleyman Yalcin City Hospital, Department of Cardiology, Istanbul – Turquia; 3 Turquia University of Health Sciences, Van Education and Research Hospital, Department of Cardiology, Van – Turquia; 4 Turquia University of Health Sciences, Istanbul Bakirkoy Dr Sadi Konuk Training and Research Hospital, Department of İnternal Medicine, Istanbul – Turquia; 5 Turquia University of Health Sciences, Kanunİ Sultan Suleiman Traİnİng and Research Hospİtal, Department of İnternal Medicine, Istanbul – Turquia; 6 Turquia University of Health Sciences, Sisli Hamidiye Etfal Training and Research Hospital, Department of Family Medicine Istanbul – Turquia; 7 Turquia University of Health Sciences, Prof Dr Cemil Tascioglu City Hospital, Department of Radiology, Istanbul – Turquia; 8 Turquia Istanbul University, Istanbul Faculty of Medicine, Department of İnternal Medicine, Istanbul – Turquia

**Keywords:** Atherosclerosis, Carotid Intima-Media Thickness, Prediabetic State

## Abstract

**Background:**

Patients with prediabetes have an increased risk of atherosclerotic cardiovascular disease; therefore, early detection is important.

**Objective:**

The present study aimed to reveal the usability of serum endocan levels as a biomarker in the diagnosis of subclinical atherosclerosis in patients with prediabetes, based on CIMT measurements.

**Methods:**

Participants were classified according to the presence (n=42) or absence (n=42) of prediabetes. Serum endocan, fasting blood sugar, fasting insulin, and glycated hemoglobin (HbA1c) values of patients were examined, and CIMT was measured. The level of significance for statistical analysis was 0.05.

**Results:**

While serum endocan levels were found to be lower in patients with prediabetes, when compared to the control group (p=0.042), CIMT values were found to be higher (p=0.046). When evaluated by multivariate regression analysis, the serum endocan level was found to be associated with CIMT, regardless of other parameters (p=0.007). A negative correlation was found between plasma fasting insulin and endocan levels (r=-0.320, p=0.001).

**Conclusions:**

Carotid intima media thickness was found to be high and the serum endocan level was low in patients with prediabetes. Decreased serum endocan levels in patients with prediabetes may be a contributing factor to atherosclerosis formation mechanisms.

## Introduction

Prediabetes, defined as levels between normal and diabetic blood sugar, is rapidly increasing around the world. Nearly 38% of the adult population in the United States of America^1^ and nearly 50% of the Chinese population have prediabetes.^2^ Prediabetes is important because of the increased risk of microvascular and macrovascular complications and progression to type 2 diabetes in a short time. High plasma glucose levels are known to be a major risk factor for atherosclerotic cardiovascular disease.^3^ Additionally, insulin resistance may be connected to atherosclerosis due to worse lipid profiles,^4^ proinflammatory state,^5^ and endothelial dysfunction.^6^

Detection of atherosclerotic cardiovascular disease in the early period is important for follow-up and treatment. Carotid intima media-thickness (CIMT) is used to detect subclinical atherosclerosis in the early stages and was able to predict cardiovascular events.^7 - 10^ Each 0.1 mm increase in CIMT increases the risk of myocardial infarction by 10-15% and stroke by 13-18%.^11^ It is very appropriate for use in large-scale population studies, as it is non-invasive and can be obtained with a simple measurement.

In addition to non-invasive methods to determine atherosclerosis development, a variety of biomarkers are known to be included in predictions. Endothelial specific molecule-1 (ESM-1), called endocan, is a proteoglycan released from endothelial cells under the control of inflammatory cytokines. Endocan activates compounds ensuring the necessary substrate for collection, adhesion, and transmigration of leukocytes along activated endothelium.^12^ In previous studies, serum endocan levels were found to be higher in patients with type 2 diabetes and acute coronary syndrome compared to control groups.^13 , 14^ Studies showed that serum endocan levels were associated with the severity of disease.^10 - 12^

There are studies assessing serum endocan levels in patients with prediabetes and insulin resistance. However, it is unclear whether changes in serum endocan levels are a cause or a consequence, particularly in atherosclerotic events. When serum endocan levels were compared in these patient groups with control groups, contrary to high values in type 2 diabetes, they were found to be low or unchanged in group with prediabetes.^15 , 16^ Although the tendency toward atherosclerosis increases in both prediabetes and diabetes patients, the differences in serum endocan levels are remarkable. There are studies evaluating endocan levels in atherosclerosis and vascular events in type 2 diabetes patients. However, we could not find any study in the literature that evaluated endocan levels in prediabetes patients over atherosclerosis.

Therefore, the present study sought to reveal the role of serum endocan levels in predicting subclinical atherosclerosis based on prediabetes patients on CIMT.

## Methods

The present study complied with the Declaration of Helsinki, and approval was obtained from the hospital research ethics committee Prof Dr Cemil Tascioglu City (approval number 525). An informed consent form was signed by the participants. This cross-sectional study was conducted in the internal medicine outpatient clinic of our tertiary care hospital between June and August 2021. A total of 84 participants over the age of 18 were included in the study, of which 42 were patients with prediabetes and 42 were normoglycemic (BMI, age, and gender are similar).

According to the American Diabetes Association (ADA) criteria, those with fasting blood glucose levels between 100-125 mg/dL (impaired glucose tolerance (IGT)) or HbA1c 5.7-6.4%, or 2nd hour plasma glucose during 75g oral glucose tolerance test (OGTT) levels between 140 and 199 mg/dL (impaired glucose tolerance (IGT)), were included in the group with prediabetes.^17^ Normoglycemic participants with lower values were included in the control group. Individuals in the normoglycemic and prediabetes groups were not using any antidiabetic drugs.

Those with a history of myocardial infarction or coronary revascularization, cerebrovascular events, a previous diagnosis of cardiovascular disease or systolic heart failure, severe valve disease, hypertrophic cardiomyopathy, angina pectoris, ST-T wave variations on electrocardiogram, Q waves, left branch block, chronic liver or kidney disease, active malignancy, hypertension, inflammatory diseases, respiratory system disease, peripheral artery disease, smokers, or those who refused to participate were excluded from the study.

The blood pressure of participants was measured, and their BMI was calculated by measuring their height and weight (weight/square of height, kg/m^2^). After overnight fasting, blood sugar, insulin HbA1c, lipid levels (high density lipoprotein cholesterol (HDL-C), low density lipoprotein cholesterol (LDL-C) and triglycerides), c-reactive protein (CRP), creatinine, and serum endocan were analyzed. Homeostasis model assessment-estimated insulin resistance (HOMA-IR) values were calculated with the formula (fasting blood sugar x fasting insulin level)/405.

### Serum endocan measurements

After overnight fasting, 10 ml of venous blood was collected from the participants. The samples were centrifuged for 10 minutes at 1700 rpm. The serum was stored at -80℃ until analysis. Serum endocan levels were measured with an enzyme-linked immunosorbent assay (ELISA) kit according to the manufacturer’s protocol (Human Endocan Elisa Kit; lot no:201506, cat. No: E3160Hu, Sunred Biological Technology Shanghai, China). Results are given as ng/L. The measurement interval of the kit is 31-2000 ng/L.

### Assessment of carotid intima-media thickness

CIMT was measured using a multifrequency (12 MHz) linear array transponder (Samsung HS50 GE Ultrasound). All measurements were taken on high-resolution B-mode images. For CIMT measurements, patients were placed in the supine position, with their heads turned 45° away from the measurement side. B-mode images of the extension of the distal segment of the right main carotid artery were obtained for three sequential sections of the far wall of the main carotid artery. The distance between the blood intima and media-adventitia interfaces was then measured for each section. CIMT was calculated by taking the average of the measurement values.

### Statistical analysis

Statistical analyses were performed using SPSS version 26.0 (SPSS Inc, Chicago, Illinois). Mean and standard deviation were used for continuous variables with normal distribution, and median and interquartile ranges were used for those without normal distribution. Categorical variables are shown as absolute numbers and percentages. Distribution of variables was assessed with the Kolmogorov–Smirnov test. Continuous variables were compared using independent two sample t-test (unpaired) or Mann-Whitney U tests according to their distribution. The chi-square test was used for categorical variables. Pearson or Spearman tests were used for correlation analysis according to whether the variables were parametric or nonparametric. Multivariate linear regression analysis was used to evaluate the CIMT determinants. Normal distribution of all parameters is required for multivariate lineer regression analysis. Normal distribution was obtained by taking the logarithm of serum endocan and triglyceride levels. The statistical significance level was determined as P < 0.05.

### Reproducibility

Considering that the intra-observer and inter-observer agreement is 0.75, the minimal sample size (assuming Type error 0.05, Type II error 0.20 Power 0.80) is n=13. Considering the possible losses for any reason, 15 people were included in the study.

### Power analysis

Power analysis was performed using the G-power program. Based on previous data in the literature, for effect size 0.57, alpha error share 5%, and 80% power to represent the population, the smallest size for each sample group was calculated as 39.

## Results

Age, gender, and BMI values of the group with prediabetes and normoglycemia were similar (p>0.05).

Serum endocan levels were significantly lower in the group with prediabetes than in the control group (p=0.042), while CIMT values were higher (p=0.046) ( Table 1 ).

**Table 1:** Demographic Features and Laboratory Findings in Patients with Prediabetes and in Controls

	Control Group n=42	Patient group with prediabetes n=42	p
Age (year)	47.8±9.7	49.9±8.5	0.112
Sex (F/M)	28/14	30/12	0.814
BMI (kg/m^2^)	33.8±4.1	32.2±8.8	0.066
Endocan (ng/L) *	138 (84-300)	120 (65-185)	0.042
FPI (µU/ml)	11.2±5.3	20.1±8.8	<0.001
FPG (mg/dL)	87±5.3	103±9.7	<0.001
2-h PG during 75-g OGTT (mg/dL)	101±19	141±34	<0.001
HOMA-IR	2.4±1.1	5.2±2.3	<0.001
HbA1c (%)	5.5 ±0.3	5.9±0.5	0.039
CRP (mg/L)	4.9± 2.6	5.1±2.9	0.245
Total Cholesterol (mg/dL)	188±32	206±33	0.020
LDL-Cholesterol (mg/dL)	110±31	120±26	0.107
HDL-Cholesterol (mg/dL)	53±11	49±13	0.103
TG (mg/dL)*	108 (79-133)	152 (95-257)	0.002
CIMT (mm)	0.67±0.16	0.74±0.17	0.046

*BMI: body mass index; FPI: fasting plasma insulin; FPG: fasting plasma glucose; PG: plasma glucose; OGTT: oral glucose tolerance test; HOMA-IR: homeostasis model assessment-estimated insulin resistance; HbA1c: glycated hemoglobin; CRP: C-reactive protein; LDL: low-density lipoprotein; HDL: high-density lipoprotein; TG: triglycerides; CIMT: carotid intima-media thickness.*

There was a significant correlation between CIMT values and age and triglyceride levels of all participants (Table-2). Multivariate lineer regression analysis of age, endocan, HbA1c, FPI, FPG, and triglyceride values was performed with CIMT. The logarithm of serum endocan and triglyceride values were taken to ensure normal distribution. The serum endocan level proved to be associated with CIMT, regardless of other parameters (p=0.007) ( Table 3 ). While there was no correlation between serum endocan levels and CIMT measurements in the group with prediabetes (r=0.104 p=0.514) ( Figure 1 ), a positive correlation was found in the group without prediabetes (r=0.340, p=0.028) ( Figure 2 ).

**Table 3:** Multivariate lineer regression analysis showing CIMT predictors

	Beta	IC 95%		p

		Inferior	Superior	
Age	0.525	0.004	0.016	0.002
FPI	0.324	-0.001	0.016	0.068
Log (TG)	-0.142	-0.381	0.154	0.396
Log (Endocan)	0.435	0.056	0.336	0.007
HbA1c	0.181	-0.053	0.219	0.222
CRP	0.024	-0.019	0.022	0.862

*FPI: fasting plasma insulin; Log: logarithm; TG: triglycerides; HbA1c: glycated hemoglobin; CRP: C-reactive protein.*

**Figure 1:** Correlation between plasma endocan levels and CIMT values in the patient group with prediabetes. (r=0.104, p=0.514)

**Figure 2:** Correlation between plasma endocan levels and CIMT values in the control group (r=0.340, p=0.028).

Correlations between the parameters in Table 1 and serum endocan levels were examined. Of these parameters, only fasting insulin was correlated with endocan levels. This correlation was negative (r=-0.320, p=0.001) ( Figure 3 ).

**Figure 3:** Correlation between serum endocan levels and fasting plasma insulin (r=-0.320, p=0.001).

### Reproducibility

A total of 15 patients were chosen at random for inter- and intra-observer variability analysis. The compatibility of intra- and inter-observer CIMT values was calculated. The intra-class correlation coefficient for intra-observer and inter-observer variability was, respectively: 0.93 (95% CI, 0.87–0.97) and 0.90 (95% CI, 0.85–0.95) for CIMT.

## Discussion

The present study attempted to explain the role of endocan levels in predicting subclinical atherosclerosis in patients with prediabetes based on CIMT measurements. Plasma endocan levels were lower in the prediabetes patient group than in the control group. By contrast, CIMT values were higher in patients with prediabetes. In our study, there was no correlation between CIMT values and serum endocan levels. When the groups were evaluated separately, the correlation between CIMT measurements and endocan levels was found in the normoglycemic group but not in the group with prediabetes. However, according to the results of the regression analysis, serum endocan levels significantly explained the CIMT value.

Many studies show that prediabetes can cause cardiovascular disease.^3 - 6^ In addition, the burden of coronary atherosclerosis in patients with prediabetes is higher than in normal people. In particular, the burden of atherosclerosis precedes the clinical symptoms of type 2 diabetes. In our study, CIMT values were high in patients with prediabetes, which is consistent with studies using CIMT as a subclinical atherosclerosis marker.^18 , 19^

Patients with prediabetes have hyperinsulinemia as a result of insulin resistance, and the results of our study are consistent with this. A negative correlation was confirmed between plasma insulin and endocan levels. It can be said that serum endocan levels are low in patients with prediabetes due to the hyperinsulinemic state.

The relationship between hyperinsulinemia and atherosclerosis has been demonstrated by previous studies. Insulin resistance has elicited great interest in medical and scientific communities because of its association with cardiovascular disease. However, the molecular mechanisms linking insulin resistance to the development and/or progression of atherosclerosis remains enigmatic. Some mechanisms come to the fore regarding this situation. Insulin signaling plays a critical role in activating nitric oxide synthase, which regulates nitric oxide production.^20 , 21^ Nitric oxide is a potent vasodilator and anti-atherogenic agent.^20^ Nitric oxide deficiency activates multiple pathways involved in atherogenesis.^22 , 23^ Thus, a defect in insulin signaling not only impairs glucose use, but also causes hypertension and accelerated atherosclerosis. It is difficult to distinguish the effect of insulin resistance from the compensatory hyperinsulinemia that always accompanies insulin resistance. It has been suggested that if the detrimental effect of insulin resistance is a result of diminished insulin action, compensatory hyperinsulinemia may be just an innocent bystander. Conversely, if certain aspects of insulin action are not affected by the decreased potency of insulin, the presence of compensatory hyperinsulinemia may have its own effect. Consequently, compensatory hyperinsulinemia can stimulate or even overstimulate certain aspects of insulin action in various cells and tissues. Thus, the primary critical point in understanding the role of insulin resistance is to determine whether reduced insulin action (effect of insulin resistance) will coexist with normal or even increased insulin action (effect of hyperinsulinemia) within the same tissue and within the same cell. This task was made possible by unraveling the intracellular insulin signaling chain. Hyperinsulinemia is a potent growth factor,^24 - 28^ whose growth-promoting effects are mediated via the mitogen-activated protein (MAP) kinase pathway.^29^ After the interaction between insulin receptor substrate-1 (IRS-1) and src homology 2 domain-containing (SHC) transforming protein, extracellular regulated kinase (ERK) is activated,^30 , 31^ translocates into the nucleus and catalyses phosphorylation of transcription factors that promote cell growth, cell proliferation, and cell differentiation.^30^ Thus, this pathway plays an important role in atherogenesis.

In addition to its role in atherosclerotic mechanisms, insulin has been reported to ameliorate the endotoxin-induced systemic inflammatory response by decreasing TNF-α expression and increasing the anti-inflammatory cascade.^26 , 32^ The expression of endocan is differentially regulated by cytokines. TNF-α and interleukin-1 beta (IL-1β) up-regulate and interferon-gamma (IFN-γ) down-regulates the secretion of endocan.^33^ The lowering effect of hyperinsulinemia on TNF-α may explain the decrease in serum endocan levels. Janke et al. also showed that endocan is expressed by human adipocytes and that insulin administration reduces endocan production in adipocytes. As a result, it has been suggested that endocan secretion from adipocytes may significantly affect local or systemic endocan levels.^34^ In our study, the suppressive effect of insulin on adipocytes may be another effective factor in the low plasma endocan levels in the patient group with prediabetes.

Menon et al. researched the role of endocan during atherosclerotic lesion formation in ApoE null mice and identified high rates of expression in atherosclerotic plaques. In the study, endocan expression was at low levels in quiescent endothelium, while they showed it was up-regulated in activated endothelium.^35^ The subjects in our study group were chosen from people without known vascular disease or any other situation causing inflammation. For this reason, there is a high probability that both controls and patients of prediabetes had quiescent endothelium. In this case, it can be said that, in our patient group, the effect of subclinical atherosclerosis on endocan secretion from the endothelium may be limited. It is our opinion that the effect of insulin on TNF-α and adipose tissue is more dominant and causes a decrease in the serum endocan level.

It has been shown that plasma endocan levels increase depending on the severity of the disease in patients with atherosclerosis, vascular inflammation, and acute coronary syndrome. This increase in the serum endocan level has proven to be associated with atherosclerotic heart diseases, but a cut-off value has not been determined.^36 , 37^ This increase in the serum endocan level has been accepted as a predictor of atherosclerosis in many studies. Endocan has been suggested as a functional inhibitor of the lymphocyte function-associated antigen 1 (LFA-1) and intercellular adhesion molecule 1 (ICAM-1) interaction, suggesting its anti-inflammatory role, through the inhibition of leukocyte rolling, adhesion, or transmigration.^12^ The beneficial effect obtained *in vivo* by blocking adhesion with mAbs in mice and in other animal models clearly demonstrates that LFA-1 and ICAM-1 are involved in acute inflammation,^38^ ischemia/reperfusion injury,^39^ allograft rejection,^40 - 42^ and antitumor immunity. Therefore, it can be said that endocan is secreted from the endothelium in response to acute inflammation and plays a regulatory role with its anti-inflammatory effect. The present study showed that serum endocan levels were decreased in patients with prediabetes, most likely due to hyperinsulinemia. It can be concluded that endocan plays an inhibitory role on the interaction between LFA-1 and ICAM-1. An increase in ICAM-1 activity is expected with decreasing endocan levels. The increase in ICAM-1 activity may cause vascular inflammation. ICAM-1 is a well-known molecule involved in the pathogenesis of atherosclerotic plaque.^43 , 44^

In studies with groups without prediabetes or insulin resistance, serum endocan levels were high, possibly in response to inflammation in the atherosclerotic vessel.^35 , 36^ However, our study showed that this response was insufficient and that serum endocan levels decreased in patients with prediabetes and subclinical atherosclerosis, especially due to hyperinsulinemia. Low serum endocan levels may be involved in atherosclerosis formation mechanisms. Comprehensive studies are needed on this subject.

### Study limitations

There are some limitations to our study. The main limitation is the low number of patients and the study being performed in a single center. Secondly, only CIMT measurements were used when assessing subclinical atherosclerosis. Finally, another limitation is that we do not know how long our patients had been prediabetic.

## Conclusions

Our results show that hyperinsulinemia causes a decrease in endocan levels. However, there is no threshold value to predict atherosclerosis. The decrease in serum endocan values measured periodically in the follow-up of patients with prediabetes may give more information about the development of atherosclerosis. Prospective studies are needed for this purpose.

**Table 2:** Correlations between CIMT and other parameters

	All participants (n=84)		Control Group (n=42)		Patient group with prediabetes (n=42)	

	r	p	r	p	r	p
Endocan (ng/L) *	0.206	0.060	0.340	0.028	0.104	0.514
Age (Year)	0.363	0.001	0.490	0.001	0.215	0.172
BMI (kg/m^2)^	-0.015	0.895	-0.009	0.956	0.034	0.833
FPI ((µU/mL)	0.180	0.104	0.360	0.021	0.020	0.900
FPG (mg/dL)	0.195	0.075	0.212	0.178	0.119	0.454
2-h PG (OGTT)	0.166	0.131	0.080	0.485	0.164	0.300
HOMA-IR	0.180	0.102	0.379	0.013	0.004	0.982
HbA1c (%)	0.242	0.080	0.349	0.143	0.199	0.260
CRP (mg/L)	0.077	0.520	0.063	0.694	0.065	0.730
Total-C (mg/dL)	-0.015	0.895	-0.076	0.632	-0.015	0.927
LDL-C (mg/dL)	-0.031	0.781	-0.093	0.557	-0.192	0.223
HDL-C (mg/dL)	-0.111	0.313	0.032	0.839	0.227	0.149
TG (mg/dL)*	0.257	0.018	0.306	0.030	0.342	0.027

BMI: body mass index; FPI: fasting plasma insulin; FPG: fasting plasma glucose; PG: plasma glucose; OGTT: oral glucose tolerance test; HOMA-IR: homeostasis model assessment-estimated insulin resistance; HbA1c: glycated hemoglobin; CRP: C-reactive protein; Total-C: Total cholesterol; LDL-C: low density lipoprotein-cholesterol; HDL-C: high density lipoprotein-cholesterol; TG: triglycerides; CIMT: carotid intima-media thickness.*Spearman correlation test, others: Pearson correlation test.
